# ADMP controls the size of Spemann's organizer through a network of self-regulating expansion-restriction signals

**DOI:** 10.1186/s12915-018-0483-x

**Published:** 2018-01-22

**Authors:** Avi Leibovich, Hadas Kot-Leibovich, Danny Ben-Zvi, Abraham Fainsod

**Affiliations:** Department of Developmental Biology and Cancer Research, Institute for Medical Research Israel-Canada, Faculty of Medicine, The Hebrew University of Jerusalem, Jerusalem, 91120 Israel

**Keywords:** Scaling, Spemann's organizer, BMP signaling, *Xenopus* embryos, dominant negative receptors, ADMP, ALK1, ALK2

## Abstract

**Background:**

The bone morphogenetic protein (BMP) signaling gradient is central for dorsoventral patterning in amphibian embryos. This gradient is established through the interaction of several BMPs and BMP antagonists and modulators, some secreted by Spemann's organizer, a cluster of cells coordinating embryonic development. Anti-dorsalizing morphogenetic protein (ADMP), a BMP-like transforming growth factor beta ligand, negatively affects the formation of the organizer, although it is robustly expressed within the organizer itself. Previously, we proposed that this apparent discrepancy may be important for the ability of ADMP to scale the BMP gradient with embryo size, but how this is achieved is unclear.

**Results:**

Here we report that ADMP acts in the establishment of the organizer via temporally and mechanistically distinct signals. At the onset of gastrulation, ADMP is required to establish normal organizer-specific gene expression domains, thus displaying a dorsal, organizer-promoting function. The organizer-restricting, BMP-like function of ADMP becomes apparent slightly later, from mid-gastrula. The organizer-promoting signal of ADMP is mediated by the activin A type I receptor, ACVR1 (also known as activin receptor-like kinase-2, ALK2). ALK2 is expressed in the organizer and is required for organizer establishment. The anti-organizer function of ADMP is mediated by ACVRL1 (ALK1), a putative ADMP receptor expressed in the lateral regions flanking the organizer that blocks expansion of the organizer. Truncated ALK1 prevents the organizer-restricting effects of ADMP overexpression, suggesting a ligand-receptor interaction. We also present a mathematical model of the regulatory network controlling the size of the organizer.

**Conclusions:**

We show that the opposed, organizer-promoting and organizer-restricting roles of ADMP are mediated by different receptors. A self-regulating network is proposed in which ADMP functions early through ALK2 to expand its own expression domain, the organizer, and later functions through ALK1 to restrict this domain. These effects are dependent on ADMP concentration, timing, and the spatial localization of the two receptors. This self-regulating temporal switch may control the size of the organizer and the genes expressed within in response to genetic and external stimuli during gastrulation.

**Electronic supplementary material:**

The online version of this article (10.1186/s12915-018-0483-x) contains supplementary material, which is available to authorized users.

## Background

Spemann’s organizer is a dynamic embryonic structure central to the establishment and patterning of the early embryo [1, 2]. The organizer secretes signals to neighboring cells regulating the size, placement, patterning, and orientation of the induced tissues and in itself undergoes morphogenesis and differentiates. In *Xenopus* embryos, one of the main roles of Spemann’s organizer is to pattern the dorsoventral axis during gastrulation, through establishment of a bone morphogenetic protein (BMP) morphogen gradient [3–8]. The organizer secretes a number of BMP antagonists and regulatory proteins to create a low level of BMP signaling on the dorsal side of the embryo, close to the organizer, and shuttle BMP molecules to the ventral side [1, 7–13]. One of the organizer-secreted BMP antagonists, chordin, has been shown to bind BMP4 and facilitate its ventral diffusion, i.e., by shuttling, contributing to the robustness and scaling of this gradient with embryo size [7, 9]. Multiple proteins have been identified that regulate the availability and stability of BMP4, including Tolloid, a metalloprotease [14], and Sizzled, a ventrally expressed and highly diffusible inhibitor of Tolloid [8, 15].

The organizer-expressed, anti-dorsalizing morphogenetic protein (ADMP), a BMP-like member of the transforming growth factor beta (TGFß) family, remains a puzzling component of the BMP signaling network [16]. ADMP is considered as a wrongly localized BMP based on its ventral-promoting effect when overexpressed [16]. ADMP also contributes to a self-regulating morphogenetic field that compensates for the loss of BMP activity [9, 17], and it negatively affects the formation of the organizer [18]. We have previously shown that dorsal ADMP expression, coupled with its BMP-like activity, may contribute to the ability to scale the BMP gradient with embryo size [9]. Several ADMP reported characteristics are inconsistent with a simple BMP-like role. Foremost, *ADMP* is robustly expressed in the dorsal region, the future organizer [16, 19], and it is a robust repressor of the organizer itself [18, 20]. ADMP is involved in the transition of the organizer, from head- to trunk-promoting, in a concentration-dependent manner [21, 22]. ADMP is also involved in a self-regulatory network involving the Nodal TGFß factors to pattern the rostrocaudal axis [22]. These observations suggest that ADMP is not a simple BMP-like ligand, and that it functions within the organizer as well as in cells outside the organizer domain.

Here, we report a novel role for ADMP in the establishment of the organizer. We show that during early gastrula (stage 10–10.25) organizer-specific gene expression is dependent on normal ADMP activity levels. We show that this activity is mediated by the dorsally localized activin A type I receptor, ACVR1 (also known as activin receptor-like kinase-2, ALK2) [19]. These observations support a novel early function for *ADMP* as a “typical” organizer gene, in agreement with its localized expression within this embryonic region. Slightly later, towards mid-gastrulation (stage 10.25–10.5), ADMP acquires an organizer-repressive activity. This anti-organizer activity is mediated by a different putative ADMP type I receptor, ACVRL1 (ALK1). ALK1 is expressed laterally [19] and mediates the organizer-repressive activity of ADMP. The ADMP signaling network was modeled to show that it can control the size of the organizer and that it relies on the availability, expression timing, and spatial localization of its receptors. A model is proposed in which ADMP signals through spatially localized receptors to determine the size of the organizer. ADMP, through ALK2, initially promotes the expansion of the organizer during its establishment. Subsequently, ADMP signals through ALK1 to restrict further expansion of this embryonic structure. The opposing ADMP activities establish a self-regulatory network that helps determine the size of the organizer domain through expansion-restriction signals and ultimately affects the BMP gradient and dorsoventral patterning of the embryo.

## Results

### ADMP has a dorsal function in Spemann's organizer

**Fig. 1:**
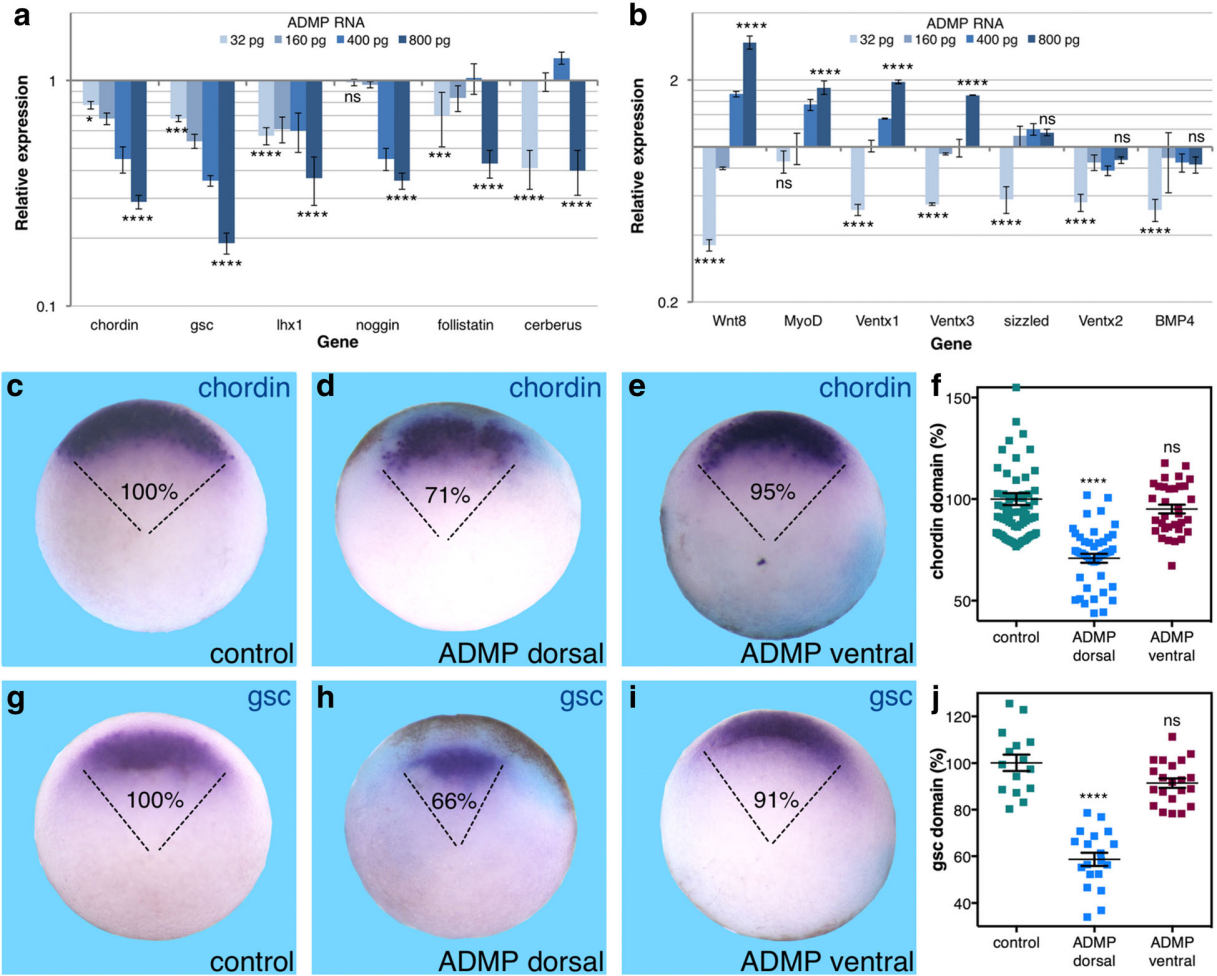
ADMP restricts the size of the organizer domain. ADMP overexpression was induced by injection of increasing amounts of capped RNA (32–800 pg/embryo). Embryos were collected during early gastrula and analyzed for changes in gene expression patterns by qPCR and in situ hybridization. **a**, **b** qPCR analysis of the expression levels of dorsal (**a**) and ventral (**b**) genes. **c**–**j** Embryos were injected in dorsal or ventral blastomeres with ADMP mRNA (100 pg/embryo) together with a lineage tracer (*turquoise*) followed by in situ hybridization analysis with *chordin* (**c**–**f**) and *gsc* (**g**–**j**) probes to determine the effect on their expression domain. The relative arc of the expression domain was determined. **f**, **j** Graphs summarizing all the measurements of the relative sizes (%) of the expression domains. *Chordin* analysis: control, *n* = 75; ADMP dorsal, *n* = 46; ADMP ventral, *n* = 33, and *gsc* analysis: control, *n* = 16; ADMP dorsal, *n* = 19; ADMP ventral, *n* = 21. Statistical test; Dunnett’s analysis of variance (ANOVA) multiple comparisons test. *p < 0.05, ***p < 0.001, ****p < 0.0001, *ns* not significant

Since its original description [16], ADMP has been a puzzling factor, as it is expressed in Spemann's organizer with an anti-organizer activity. To better understand the function of ADMP as a putative BMP during early embryogenesis, we studied its regulatory effects when overexpressed at different levels (32–800 pg injected messenger RNA, mRNA). Embryos injected with increasing amounts of ADMP*-*capped RNA were analyzed during early gastrula (stage 10–10.25) [23]. The effect on the expression of ventral and dorsal genes was determined by quantitative real-time (RT)-polymerase chain reaction (PCR), hereafter called qPCR. Expression of organizer genes like *gsc* and *chordin* exhibited a dose-dependent down-regulation by all amounts of ADMP mRNA injected (Fig. 1a), while genes like *lhx1* (*Xlim1*) and *noggin* exhibited a repressive threshold response. For most organizer genes, any increase above the endogenous ADMP levels in the early organizer results in reduced transcript levels.

The reduction in dorsal-specific gene expression could stem from lower transcription within the organizer or a contraction of the expression domain, i.e., a reduction of the organizer. To better understand the repression of dorsal gene expression, we injected ADMP mRNA in dorsal or ventral blastomeres together with a lineage tracer to verify the injection site. Embryos analyzed during early gastrula (stage 10.25) exhibited a contraction in the domain of c*hordin* and *gsc* expression (Fig. 1c–j). To quantitate the reduction in organizer size, the arc of the *chordin* and *gsc* expression domains was measured. Due to the dynamic nature of the expression patterns of the organizer genes, care was taken to compare embryos at the same stage, and the arc in the experimental embryos is shown as a change from the arc in control embryos (%). Dorsal ADMP overexpression efficiently reduced the c*hordin* expression domain by 29% compared to that in the control embryos (Fig. 1c, d, f). Interestingly, injection of ADMP RNA to ventral blastomeres had no significant effect on the *chordin* expression domain (95% arc; Fig. 1e, f). Similarly, dorsal ADMP reduced the *gsc* expression domain by 34% from the control size, while ventral overexpression had a weak, not statistically significant effect (91% arc; Fig. 1g–j). These results show that high ADMP levels reduce the size of the organizer, but this effect is almost absent when the RNA encoding this protein is injected far from the organizer in the ventral half of the embryo.

To determine the effect of *ADMP* overexpression on genes normally expressed in the lateral and ventral marginal zones, the RNA samples from embryos expressing increasing amounts of ADMP mRNA were analyzed. qPCR analysis for a series of ventrolateral genes and BMP target genes, including *BMP4* itself, revealed that, surprisingly, low *ADMP* levels repressed the expression of the genes studied by about 50% (Fig. 1b). Increasing the amount of ADMP RNA injected shifted the effect to the expected, ventral-promoting effect, resulting in up-regulation of some of the ventral markers (Fig. 1b; *Wnt8*, *MyoD*, *Ventx1*, and *Ventx3*), while genes like *sizzled*, *Ventx2*, and *BMP4* were not up-regulated even by high ADMP levels. These results show that ADMP exerts a reversing, concentration-dependent, regulatory role on genes along the dorsoventral axis.

**Fig. 2:**
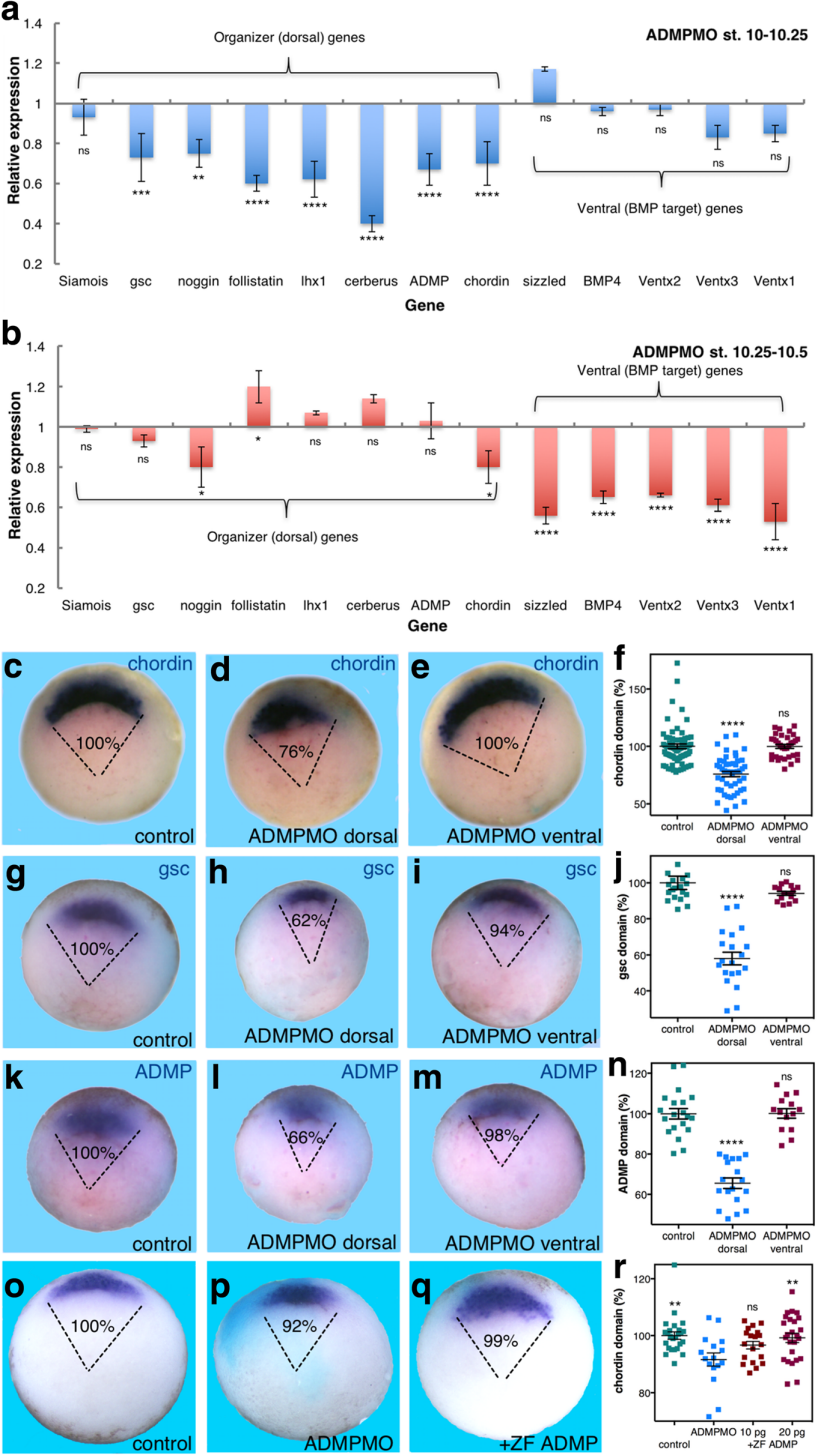
ADMP provides dorsal signal required in Spemann's organizer at the onset of gastrulation. ADMP knockdown was induced by ADMPMO injection (7 ng/embryo) and subjected to analysis of changes in gene expression. **a**, **b** qPCR analysis of the effects on dorsal and ventral gene expression during early gastrulation (stage 10–10.25) (**a**) and later during early/mid-gastrula (stage 10.25–10.5) (**b**). **c**–**n** Effect of ADMPMO injection on the domains of *chordin* (**c**–**f**), *gsc* (**g**–**j**), and *ADMP* (**k**–**n**) expression. Embryos injected with ADMPMO (4 ng) either dorsally or ventrally were fixed at the onset of gastrulation. **f**, **j**, **n** The arc of the domain of expression was measured, and the size of the domain of expression relative to control embryos is shown (%). **o**–**r** The specificity of the ADMPMO was further tested in rescue experiments. Embryos were injected dorsally (*turquoise*) with ADMPMO (3.4 ng/embryo) alone or with increasing amounts of RNA encoding the zebrafish (ZF) ADMP protein (10 or 20 pg RNA/embryo). **o**–**q** In situ hybridization analysis of the changes in the *chordin* expression domain in control (**o**), ADMPMO injected (**p**), and embryos co-injected with ADMPMO and zebrafish ADMP mRNA (20 pg/embryo) (**q**). **r** Summary of the quantitation of the changes in the size of the *chordin* expression domain. *Chordin* analysis: control, *n* = 81; ADMPMO dorsal, *n* = 48; ADMPMO ventral, *n* = 33, *gsc* analysis: control, *n* = 19; ADMPMO dorsal, *n* = 20; ADMPMO ventral, *n* = 15, *ADMP* analysis: control, *n* = 20; ADMPMO dorsal, *n* = 18; ADMPMO ventral, *n* = 14; and *chordin* rescue analysis: control, *n* = 25; ADMPMO, *n* = 25; ADMPMO + ZF ADMP 10, *n* = 19; ADMPMO + ZF ADMP 20, *n* = 25. *p* values were calculated compared to controls (**a**, **b**, **f**, **j**, **n**) except in the rescue experiments that were compared to the ADMPMO sample (**r**). Statistical test; Dunnett’s (ANOVA) multiple comparisons test. *p < 0.05, **p < 0.01, ***p < 0.001, ****p < 0.0001, *ns* not significant

The early repression of ventrolateral gene expression could be interpreted as a weak, dorsal-promoting effect of ADMP. To obtain better evidence of a dorsal ADMP function, we focused on knockdown using the previously characterized ADMP antisense morpholino oligonucleotide (ADMPMO) [17]. ADMP knockdown avoids the possible complications of gain of function and the widely accepted functional overlap with BMP members. Dorsal- and ventral-specific gene expression was studied in morphants and control embryos from late blastula to mid-gastrula. Analysis during early gastrula (stage 10–10.25) [23] revealed that ADMP knockdown significantly reduced organizer-specific gene expression ranging from about 30 to 60% for *gsc*, *noggin*, *follistatin*, *lhx1*, *cerberus*, *chordin*, and *ADMP* itself, but hardly affected the immediate early organizer gene *Siamois* (Fig. 2a). At this stage, ventral BMP target genes like *Ventx1* (*Vent1*), *Ventx2* (*Vent2*), *Ventx3* (*Vex1*), *BMP4*, and *sizzled* were marginally affected (Fig. 2a). Slightly later during gastrulation (stage 10.25–10.5), *ADMP* morphants exhibited the reverse effect, such that BMP target gene expression showed strong and significant transcript reduction by about 30–50% for *BMP4*, *sizzled*, *Ventx1*, *Ventx2*, and *Ventx3* (Fig. 2b). In contrast, the ADMP requirement for organizer-specific gene expression at this later stage was very weak (Fig. 2b). ADMP loss of function uncovered a previously unknown and very early requirement for this factor in the establishment of normal organizer gene expression. Once gastrulation is well underway, ADMP shifts to its accepted ventral-promoting, BMP-like role.

To study the effect of ADMP knockdown on organizer size, we injected embryos dorsally or ventrally with ADMPMO. The embryos were analyzed for changes in organizer-specific expression domains at the onset of gastrulation. The decrease in RNA levels observed by qPCR in *ADMP* morphants reflects a significant narrowing of the organizer domain, while the signal intensity remained similar among samples. Dorsal ADMP knockdown resulted in a statistically significant reduction in the *chordin* expression domain by about 24% (Fig. 2d, f). Embryos injected ventrally exhibited no significant change in the *chordin* expression domain (100%; Fig. 2e, f). Analysis of the effect on *ADMP* and *gsc* expression also showed a significant narrowing by 34% and 38% respectively (Fig. 2g–n), whereas ventral ADMPMO injection resulted in almost normal expression domains (98% and 94% respectively; Fig. 2i, m). Dorsal co-injection of RNA encoding the zebrafish (ZF) ADMP protein [24] rescued the size of the organizer in embryos injected dorsally with ADMPMO in a concentration-dependent manner, supporting the specificity of the knockdown (Fig. 2o–r). Also in these experiments, dorsal knockdown of the ADMP activity resulted in significant narrowing of the organizer as monitored by the *chordin* expression domain (Fig. 2p). These results demonstrate that the main effect of dorsal ADMP knockdown at the onset of gastrulation is a decrease in the size of the organizer, supporting an early ADMP dorsal function in the establishment of a normal-sized organizer domain.

### ALK2 mediates the dorsal, organizer-promoting function of ADMP

**Fig. 3:**
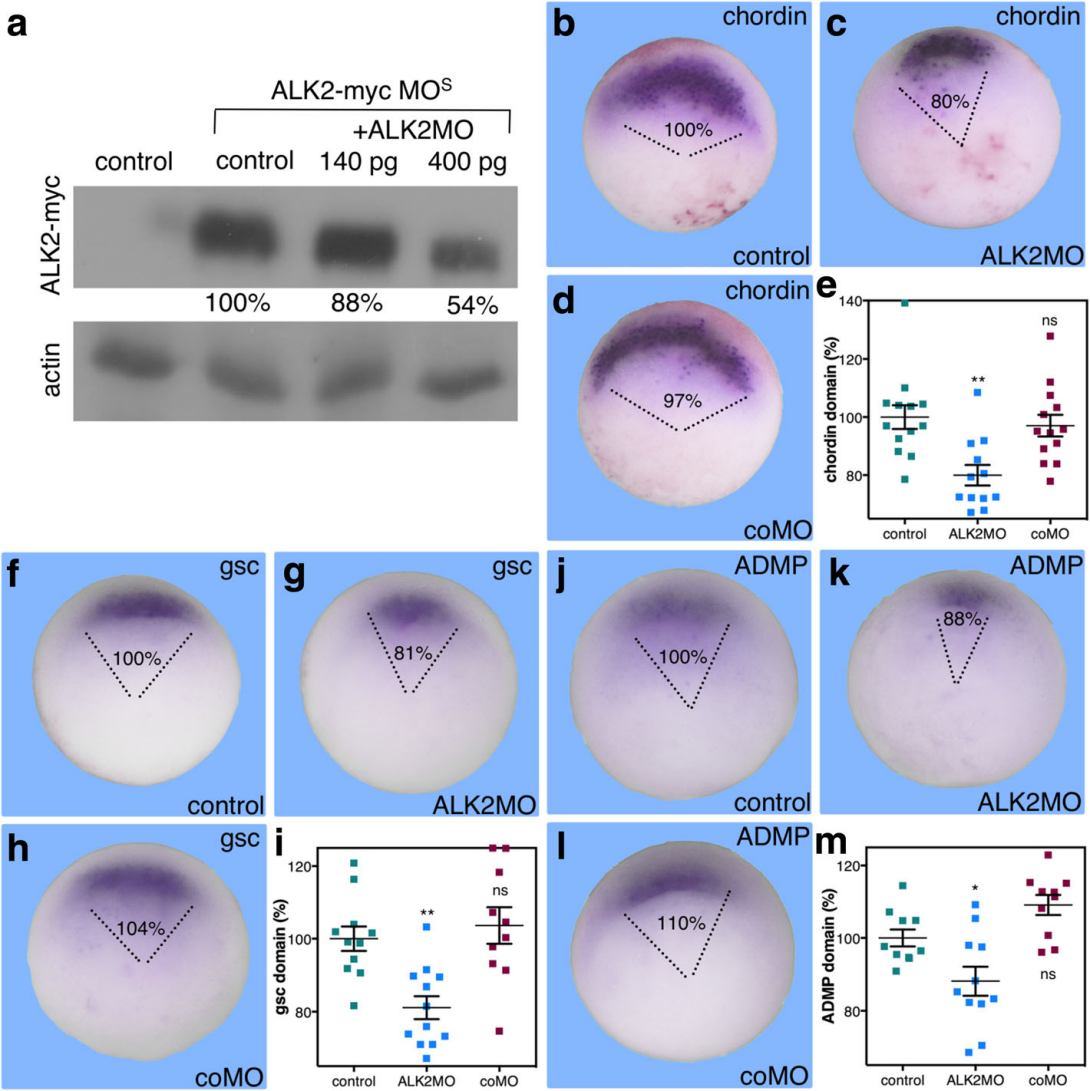
ALK2 is required for the expansion of the organizer domain. **a** The efficacy of the ALK2MO was determined by western blot analysis. RNA encoding a myc-tagged version of the ALK2 protein with the ALK2MO recognition sequence was co-injected into embryos with increasing amounts of ALK2MO. Early gastrula protein extracts were subjected to electrophoresis and immunodetection with the 9E10 anti-myc monoclonal antibody. To control the loading and transfer steps, the blot was stripped and re-probed with an anti-actin antibody. **b**–**m** Embryos were injected with an antisense morpholino oligonucleotide specific for ALK2 (ALK2MO; 400 pg/embryo) or control MO (coMO). At the onset of gastrulation, embryos were analyzed by in situ hybridization with *chordin* (**b**–**e**), *gsc* (**f**–**i**), and *ADMP* (**j**–**m**) specific probes to determine the effect on their expression domain. The domain of expression was determined by measuring the arc in degrees and calculating the relative size compared to control embryos. The relative (%) arc of the expression domain was determined. *Chordin* analysis: control, *n* = 13; ALK2MO, *n* = 12; coMO, *n* = 13, *gsc* analysis: control, *n* = 11; ALK2MO, *n* = 12; coMO, *n* = 10, and *ADMP* analysis: control, *n* = 9; ALK2MO, *n* = 10; coMO, *n* = 8. *p* values were calculated compared to controls. Statistical test; Dunnett’s (ANOVA) multiple comparisons test. *p < 0.05, **p < 0.01, *ns* not significant

ADMP has been shown to bind the type I receptor, ACVR1 (ALK2) [17], while the common BMP receptors, BMPR-Ia (ALK3) and BMPR-Ib (ALK6) and the type II receptor, BMPR-II, do not bind ADMP or inhibit its activity [17, 21]. We have recently shown that *Alk2* is expressed within the organizer and it is co-expressed with *ADMP* [19]. As the putative, dorsal ADMP receptor, ALK2 should be involved in the organizer-promoting function of ADMP (Figs. 1 and 2). To support this suggestion, we designed an antisense morpholino oligonucleotide (MO) to knockdown the ALK2 activity (ALK2MO). The morpholino was tested by western blot analysis against a myc-tagged version of the ALK2 protein preceded by the ALK2MO homology sequence. In embryos co-injected with ALK2-myc mRNA and ALK2MO, translation of the ALK2-myc protein was inhibited by ALK2MO in a concentration-dependent manner (Fig. 3a).

**Fig. 4:**
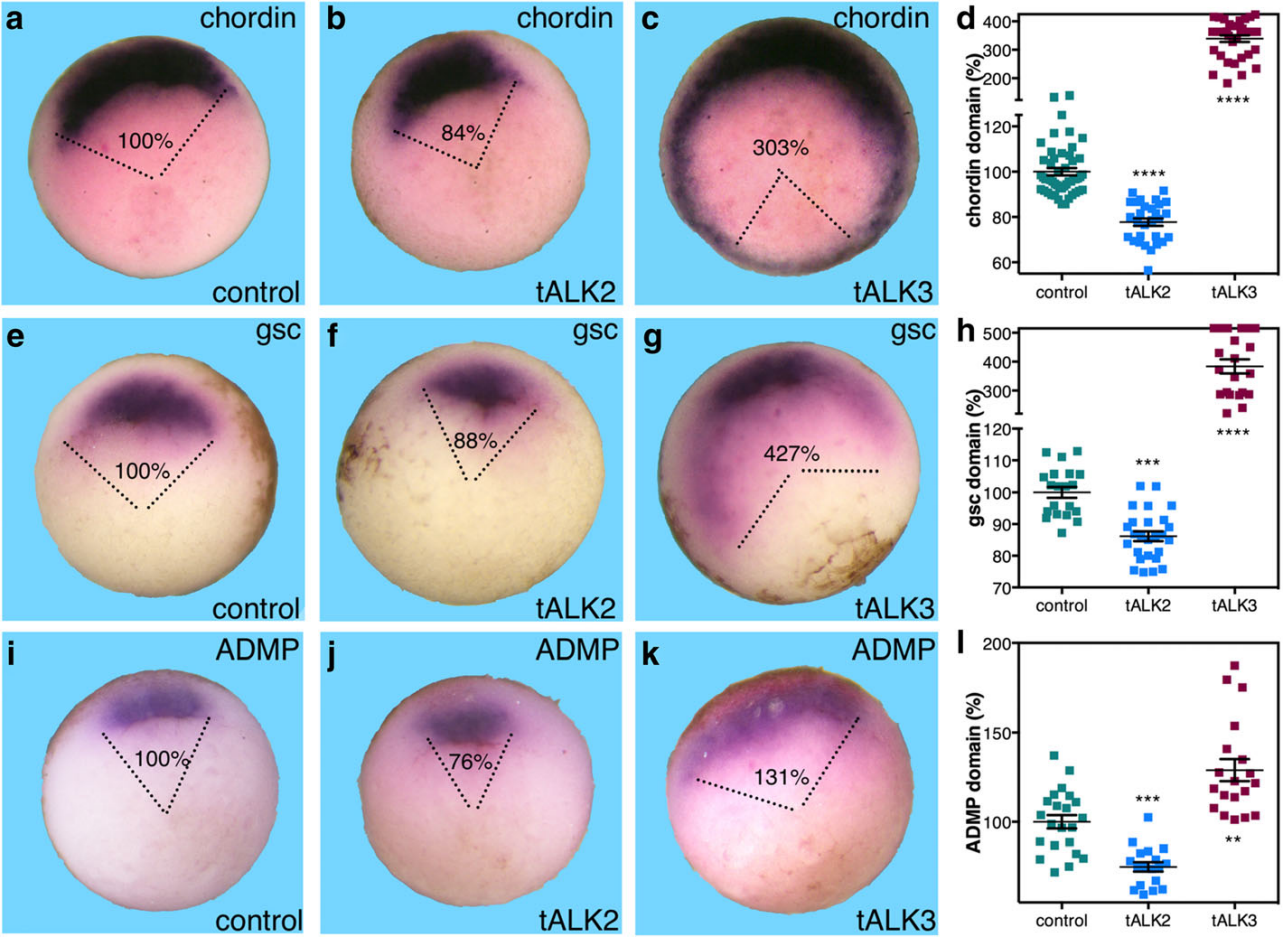
The ALK2 receptor positively regulates the expression of organizer genes. The truncated, dominant negative form of ALK2 (tALK2) was employed to corroborate the function of this receptor in the expansion of the organizer. Embryos were injected with mRNA (1.3 ng/embryo) encoding tALK2 or the truncated ALK3 (tALK3) in order to block their activity. At the onset of gastrulation, the effect of blocking these receptors on the expression domains of *chordin* (**a**–**d**), *gsc* (**e**–**h**), and *ADMP* (**i**–**l**) was determined by in situ hybridization with specific probes. The relative size of the expression domain (%) was determined. *Chordin* analysis: control, *n* = 28; tALK2, *n* = 20; tALK3, *n* = 15, *gsc* analysis: control, *n* = 20; tALK2, *n* = 26; tALK3, *n* = 23, and *ADMP* analysis: control, *n* = 10; tALK2, *n* = 18; tALK3, *n* = 19. *p* values were calculated compared to controls. Statistical test; Dunnett’s (ANOVA) multiple comparisons test. **p < 0.01, ***p < 0.001, ****p < 0.0001

Early gastrula embryos (stage 10.25) injected with ALK2MO were analyzed to determine the effect on the domains of the dorsal genes *chordin*, *gsc*, and *ADMP* (Fig. 3b–m). Reduction in the level of ALK2 activity resulted in significant reduction in the size of the expression domains of *chordin*, *gsc*, and *ADMP* to 80%, 81%, and 88% respectively (Fig. 3c, g, k). Also in this case, the signal intensity remained comparable between manipulations. Similar to the loss of ADMP activity, ALK2 knockdown hinders the formation of the organizer and compromises its size. To corroborate the ALK2MO results, we generated a truncated, dominant negative form of ALK2. The truncated ALK2 (tALK2) was constructed by deleting the intracellular kinase domain as described for ALK3 [25, 26] (see Methods). The effect of tALK2 on dorsal gene expression was studied during early gastrula (stage 10.25) and compared to tALK3, which blocks BMP4 signaling [25, 26]. Injection of tALK2 RNA caused a significant reduction in the domain of *chordin* expression to 84% of the size of the control expression domain (Fig. 4b, d). In contrast, overexpression of tALK3 resulted in a marked expansion of the *chordin* expression by more than 200% (Fig. 4c, d). Similarly, tALK2 overexpression reduced the domains of *gsc* to 88% (Fig. 4f, h) and *ADMP* to 76% (Fig. 4j, l). The domains of *gsc* and *ADMP* also increased as a result of blocking the BMP signal with tALK3 to 427% and 131% respectively (Fig. 4g, h, k, l). The tALK2-induced changes in the expression domains of organizer genes corroborate the results obtained with ALK2MO. These results also show that ALK2 and ALK3 mediate opposed functions along the dorsoventral axis, supporting the suggestion that ALK2 performs a dorsal activity required to achieve the normal organizer size.

### ALK1 functions in the lateral restriction of organizer expansion

The organizer-restricted expression of *Alk2* prompted us to identify the type I receptor that mediates the BMP-like activity of ADMP. ALK1, ALK2, ALK3, and ALK6 are known to be the main type I receptors mediating BMP and GDF signaling [27]. Therefore, we focused on ALK1 (ACVRL1) as a putative second ADMP receptor. Recently we described the *Alk1* expression pattern during early gastrula [19]. *Alk1* is a weakly expressed gene during gastrula stages, and its transcripts are most abundant in the lateral marginal zone (LMZ), just flanking the organizer domain. This distribution is consistent with ALK1 being localized to mediate the BMP-like activity of ADMP, once it diffuses out of the organizer.

**Fig. 5:**
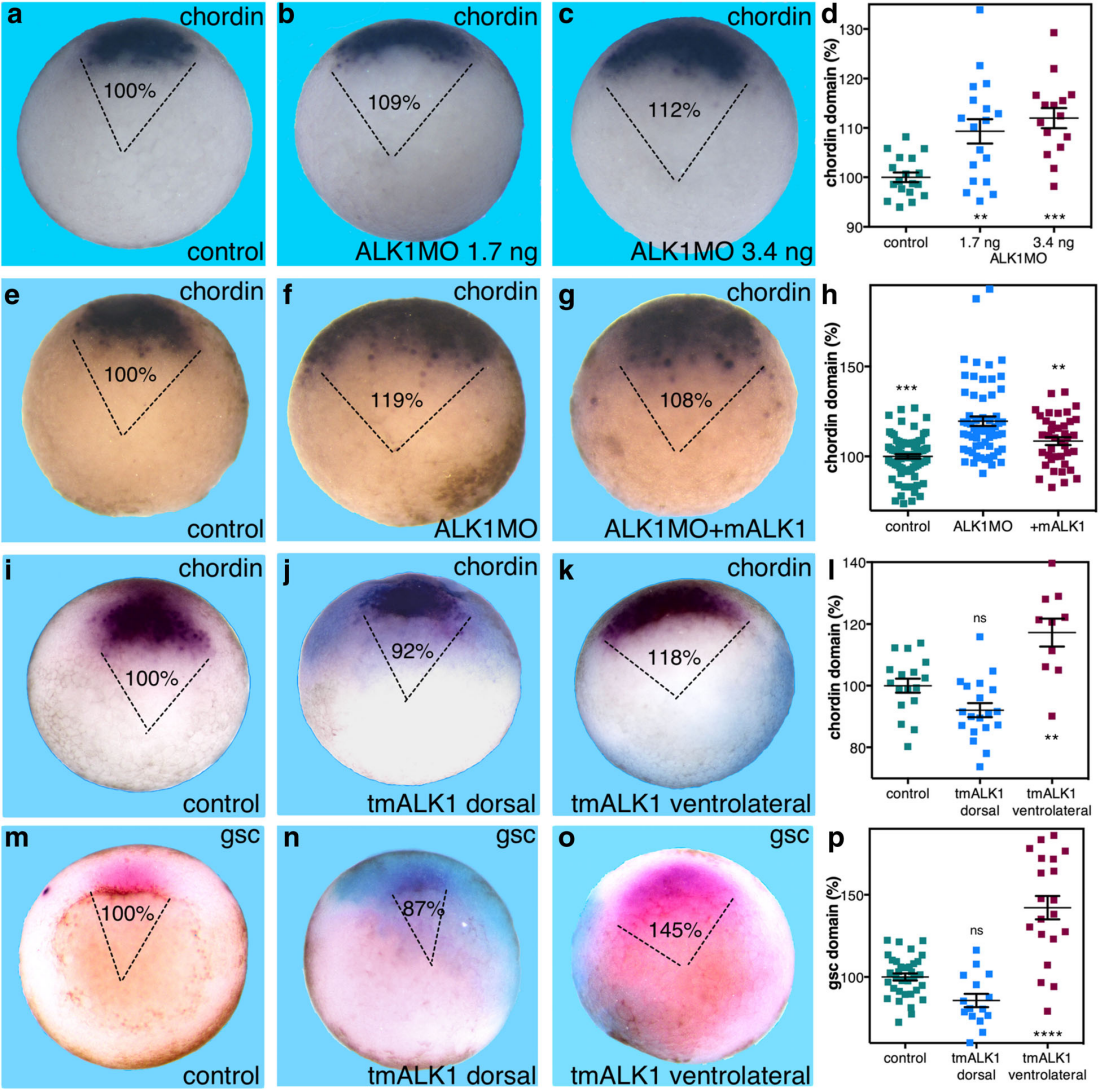
ALK1 is required to restrict the size of the organizer domain. **a**–**d** To determine the function of the ALK1 receptor during early gastrulation, embryos were injected with increasing amounts of ALK1MO (3.4 and 1.7 ng/embryo). The effect of blocking this receptor on the *chordin* expression domain was determined by in situ hybridization. The relative (%) arc of the expression domain was determined. **e**–**h** The specificity of the ALK1MO (1.7 ng/embryo) was analyzed by co-injection with RNA encoding the mouse ALK1 receptor (2.2 ng/embryo). Changes in the domain of *chordin* expression were quantitated. **i**–**p** RNA encoding the truncated, dominant negative form of the mouse ALK1 receptor (tmALK1) was injected into *Xenopus* embryos either in the dorsal or ventrolateral regions of the embryo. The effect of the ALK1 activity was determined by in situ hybridization. Analysis of the changes in the *chordin* (**i**–**l**) and *gsc* (**m**–**p**) expression domains was performed at the onset of gastrulation. The relative (%) arc of the expression domain was determined in each case. ALK1MO amounts analysis: control, *n* = 18; ALK1MO 1.7, *n* = 18; ALK1MO 3.4, *n* = 15, ALK1MO rescue analysis: control, *n* = 83; ALK1MO, *n* = 62; ALK1MO + mALK1, *n* = 40, tmALK1 *chordin* analysis: control, *n* = 17; tmALK1 dorsal, *n* = 19; tmALK1 ventrolateral, *n* = 10, and tmALK1 *gsc* analysis: control, *n* = 34; tmALK1 dorsal, *n* = 15; tmALK1 ventrolateral, *n* = 20. *p* values were calculated compared to controls except in the rescue experiments that were compared to the ALK1MO sample (**h**). Statistical test; Dunnett’s (ANOVA) multiple comparisons test. **p < 0.01, ***p < 0.001, ****p < 0.0001, *ns* not significant

To study the role of ALK1 in the establishment of Spemann's organizer, we designed a morpholino oligonucleotide (MO) against the *Xenopus laevis* transcript of this receptor (ALK1MO). Characterization of ALK1MO by injecting increasing amounts revealed a concentration-dependent, statistically significant expansion of the *chordin* expression domain (Fig. 5a–d). This observation suggests that ALK1 performs an anti-organizer function preventing the expansion of this embryonic region. Co-injection of ALK1MO together with RNA encoding the mouse ALK1 receptor (mALK1) partially rescued the ALK1MO-mediated expansion of the *chordin* domain (Fig. 5e–h). These rescue results support the specificity of ALK1MO and further support the role of ALK1 in preventing the expansion of the organizer domain.

**Fig. 6:**
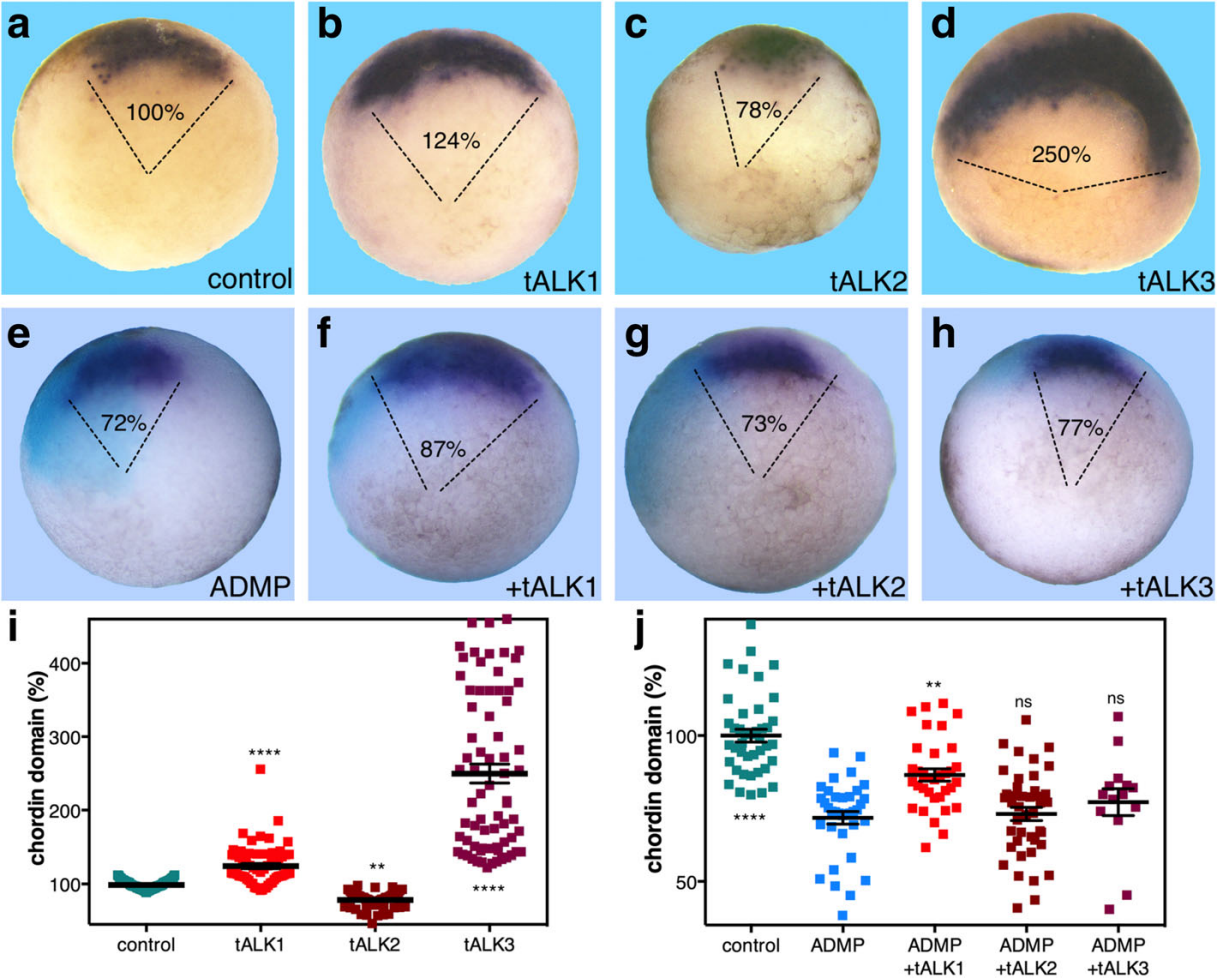
Blocking the ALK1 receptor preferentially blocks the anti-organizer activity of ADMP. **a**–**d**, **i** Embryos were injected with RNA encoding the tALK1, tALK2, and tALK3 dominant negative receptors. During early gastrula stages the changes in the *chordin* expression domain were determined by in situ hybridization. **e**–**h**, **j** To determine the receptor mediating the organizer-repressive activity of ADMP, four-cell-stage embryos were injected in a single dorsal blastomere with ADMP mRNA (50 pg/embryo) alone or together with one of the dominant negative type I receptors, tALK1, tALK2, or tALK3 (320 pg/embryo). Also, fluorescein isothiocyanate (FITC)-dextran was included as a lineage tracer (*turquoise*). At the onset of gastrulation, the changes in the size of the *chordin* expression domain were studied. The embryos were analyzed for the expression domain changes induced by ADMP gain of function and the extent of rescue by the different dominant negative receptors. The relative (%) arc of the expression domain was determined. Comparative truncated receptor analysis: control, *n* = 24; tALK1, *n* = 24; tALK2, *n* = 15; tALK3, *n* = 21, and block ADMP ventral activity analysis: control, *n* = 18; ADMP, *n* = 21; +tALK1, *n* = 16; +tALK2, *n* = 21; tALK3, *n* = 14. *p* values were calculated compared to controls (**i**) except in the rescue experiments that were compared to the ADMP sample (**j**). Statistical test; Dunnett’s (ANOVA) multiple comparisons test (**j**) and two-tailed *t* test (**i**). **p < 0.01, ****p < 0.0001, *ns* not significant

Support for the role of ALK1 in restricting the size of Spemann's organizer was obtained using a truncated, dominant negative form of this receptor (tALK1). RNA encoding tALK1 was injected radially into embryos, and during early gastrula the changes in the *chordin* expression domain were determined. For comparison, we overexpressed in parallel either tALK2 or tALK3. Overexpression of tALK1 resulted in significant expansion of the *chordin* expression domain to 124% from the control size (Fig. 6a, b, i). This alternative approach to block the ALK1 activity recapitulates the outcome of ALK1MO injection (Fig. 5). Blocking the ALK2 activity with tALK2 resulted in a reduction of the *chordin* expression domain to 78% of the control size (Fig. 6c, i), a reversal of the effect of tALK1, supporting the specificity of the truncated receptors. As previously observed, tALK3 overexpression induces a strong (up to 250%) expansion of the organizer as evidenced by the *chordin* expression domain (Fig. 6d, i). These results support the suggestion that ALK1 mediates the anti-organizer activity of ADMP, preventing the expansion of the organizer during early gastrula.

To further corroborate the specificity of the effect observed, a truncated form of the murine ALK1 receptor was also constructed (tmALK1). tmALK1 mRNA was injected dorsally or ventrolaterally as evidenced by the lineage tracer (Fig. 5i–p). Analysis of *chordin* expression revealed that dorsal tmALK1 overexpression resulted in a minor but not statistically significant change when compared to control embryos (92% and 100% respectively; Fig. 5i, j, l). However, ventrolateral injection of tmALK1 RNA resulted in a significant expansion of the *chordin* expression domain to 118% (Fig. 5k, l). Ventrolateral tmALK1 overexpression also induced significant enlargement of the *gsc* expression domain to 145%, while dorsal injection had a minor and not significant effect (Fig. 5m–p). These observations show that tmALK1 can inhibit the activity of the endogenous *Xenopus* ALK1 receptor, like tALK1, with the same outcomes. From these results, we conclude that ALK1 in the LMZ restricts the size of Spemann's organizer and prevents its lateral expansion.

Our results suggest that ADMP functions through ALK1 to perform its BMP-reminiscent, anti-organizer activity that restricts the size of the organizer domain. Therefore, inhibiting ALK1 should prevent the organizer-restricting function of ADMP. To test this hypothesis, embryos were injected with ADMP mRNA in one of the two dorsal blastomeres at the four-cell stage to induce a narrowing of the organizer. The ADMP RNA was co-injected with one of the three truncated receptors: tALK1, tALK2, or tALK3. Using *chordin* expression to monitor the changes in organizer size, we found that ADMP overexpression efficiently reduced the expression domain (72%) compared to that of the control embryos (Fig. 6e, j). Co-injection of tALK1 RNA partially rescued the organizer from reduction to 87% (Fig. 6e, f, j), while co-injection of tALK2 or tALK3 RNA had no rescuing effect on the ADMP-induced organizer narrowing (73% and 77% respectively; Fig. 6e, g, h, j). These results support the model suggesting that ALK1 functions with ADMP in the LMZ to restrict the expansion of the organizer.

### Spatio-temporal interaction between ALK1, ALK2, and ADMP: mathematical model

To better understand the relationship between ADMP and its two putative receptors, ALK1 and ALK2, we constructed a mathematical spatio-temporal model that describes the interactions between this ligand and its two receptors as a function of time in the short period from the onset of gastrulation to stage 10.25 (Additional file 1: Mathematical model). The model focused specifically on testing whether a system in which ADMP induces the organizer in one domain and represses it in another confers robustness to the ADMP production rate, and whether it has an advantage over the simple, induction-only model, where ADMP induces the organizer above a certain signal threshold in a classic morphogen fashion. Our model directly addresses the robustness of the dual function of ADMP in organizer formation and does not aim to explain other aspects in organizer establishment such as scaling of organizer size with embryo size. We employed a reaction-diffusion one-dimensional model to describe the interaction of ADMP with ALK1 and ALK2 in dorsoventral patterning. For simplicity, we did not include the binding of other TGFß ligands to these receptors or the interaction of ADMP with extracellular inhibitors such as chordin, noggin, and follistatin.

**Fig. 7:**
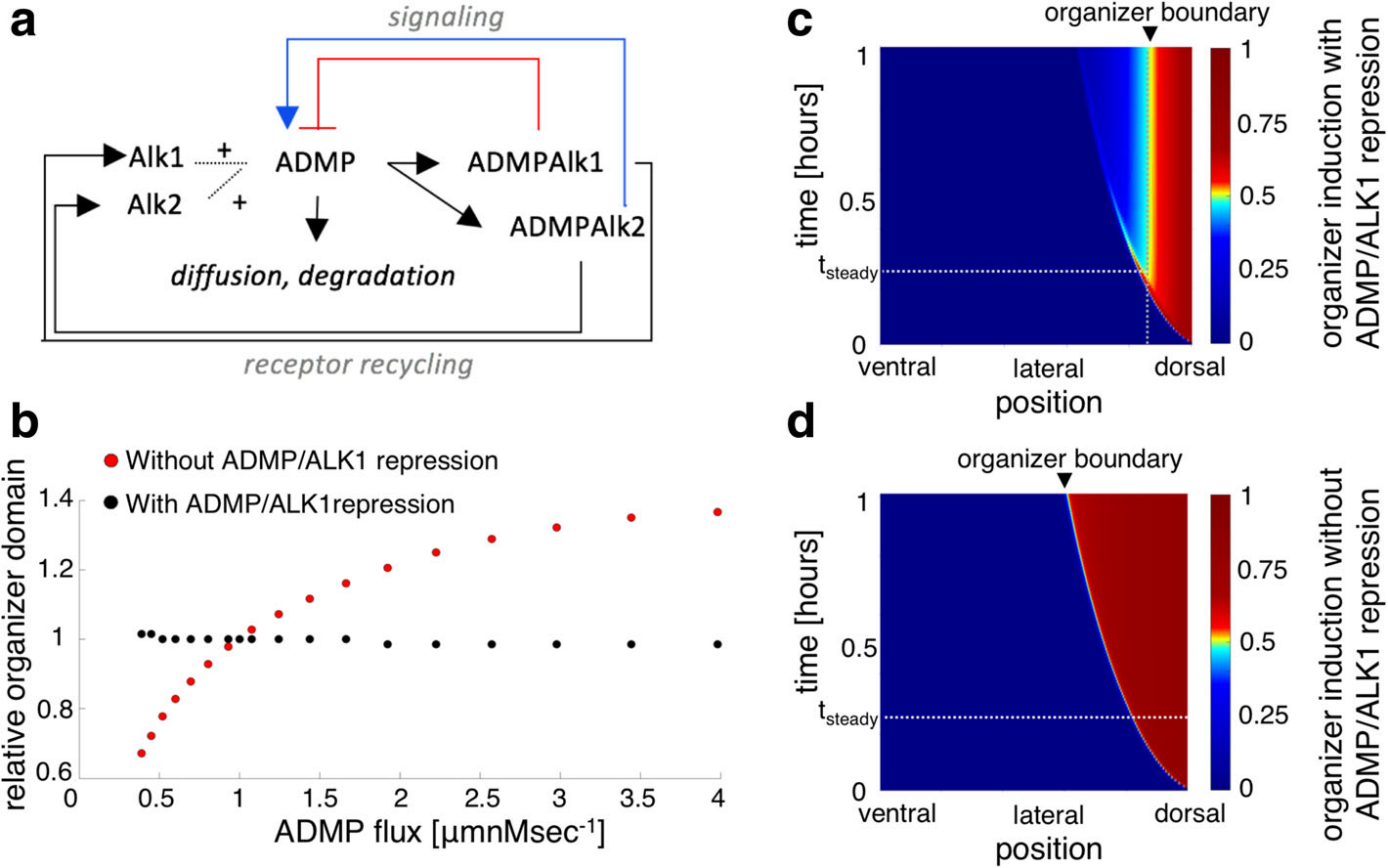
Mathematical model for the interaction between ALK1, ALK2, and ADMP. **a** Model design: ADMP can diffuse, degrade, or bind ALK1 or ALK2 receptor. The occupied receptors then induce (ADMP/ALK2) or repress (ADMP/ALK1) the expression of *ADMP* and other organizer genes. ADMP is degraded after binding the receptor, and the receptor is recycled back to the surface. **b** Size of the organizer induction domain at the end of simulation, *t* = 1 h, as a function of ADMP flux relative to its size for the reference parameter set where ADMP flux is 1 μmnM s^–1^. The organizer size is robust to ADMP flux in the dual receptor model where ALK1 represses organizer induction (*black circles*), while size is sensitive and continues to increase in a model that does not include ADMP/ALK1-mediated organizer repression (*red circles*). **c**, **d** Heat map of the organizer induction domain (θ), where 1 denotes full induction, and 0 no induction in the two-receptor model (**c**) and in a classic morphogen model, with no repression by ADMP/ALK1 (**d**). *X* axis denotes position along the dorsoventral axis, *dashed vertical gray line* denotes the steady state organizer induction domain. *Y* axis denotes time. *t*_steady_ is the time when steady state organizer induction is achieved in the two-receptor model (**c**). The organizer induction domain does not reach steady state in the simulation time in the absence of ADMP/ALK1 repression (**d**)

ALK2 is a dorsally localized receptor, while ALK1 is expressed mostly in lateral and ventral regions [19]. Induction of the organizer domain occurs where ALK2 but not ALK1 is occupied by ADMP. As part of the model, ADMP can diffuse or degrade, or it can bind the ALK1 and ALK2 receptors (Fig. 7a and Additional file 1: Mathematical model). We assume that once ADMP is bound to a receptor, it will be internalized and degraded, while the receptor is recycled to the surface. Hence the total number of receptors, occupied or free, is assumed to be constant in space and time, and we do not include regulation of ADMP over the expression of either receptor. We assume that internalization of the bound receptor is faster than ligand-receptor dissociation and did not model the dissociation of the complex. We model ADMP production by applying a constant flux of ADMP from the dorsal pole.

Numerical analysis of the model had two results. First, the induction of the organizer domain was robust to changes in the flux of ADMP (Fig. 7b). This is evident by obtaining the same organizer induction size even though the flux of ADMP changes by almost an order of magnitude. The alternative, the induction-only model, in which ADMP acts as a classic morphogen inducing the organizer domain via ALK2 signaling, was not robust to changes in ADMP flux (Fig. 7b). Intuitively, this robustness is achieved because higher ALK2 signaling caused by increased levels of ADMP leads at the same time to higher levels of repressive ALK1 signaling. As expected, the exact size of the organizer domain is sensitive to the initial distribution of the receptors, the thresholds of induction and repression of ADMP, and the binding rate to the receptors. However once these parameters are fixed, according to the model, the organizer boundary remains insensitive to ADMP flux (Additional file 2: Figure S1).

The second result, perhaps less intuitive, is that a steady state of organizer induction domain is achieved very fast (Fig. 7c), while the ADMP profile does not reach a steady state profile in our simulations (Additional file 3: Figure S2C). Moreover, the ADMP/receptor complexes also do not reach a steady state in our simulations (Additional file 3: Figure S2A, B). The organizer induction domain remains stable over a wide range of ligand/receptor binding parameters (Additional file 3: Figure S2D–F). In comparison, the organizer induction domain continues to expand in our simulation of the induction-only model (Fig. 7d). This result is relevant, since development is very rapid at these stages, and gradient profiles may not reach a steady state.

## Discussion

### Dual ADMP functions

Since its original description, it has been difficult to reconcile the organizer expression of *ADMP* with its BMP-like, organizer-repressive activity. ADMP can ventralize the embryo when overexpressed [16], suppress the expansion of the organizer [16, 18, 20], contribute to the BMP morphogen gradient, and actually compensate for it in embryos lacking a BMP signal [17, 18, 24]. Previously we showed that the BMP-like function of ADMP, coupled with its dorsal expression, could contribute to scaling of the BMP morphogen gradient [9]. ADMP also can contribute to the anterior-posterior patterning of the embryo, promoting the transition from the head- to trunk-organizer during gastrulation [18, 21, 22, 24]. In addition, ADMP competes with the organizer Nodal signals for the limited availability of their type II receptor [22]. These functions require an ADMP activity within the organizer. We show that during early gastrula (stage 10–10.25) ADMP has an organizer-promoting role and is required for the establishment of the normal organizer gene network. In contrast, analysis of ADMP knockdown slightly later, during early/mid-gastrula (stage 10.25–10.5), showed a regulatory effect centered on the ventral genes. At these later stages, the effect on organizer-specific expression was weak at best and could be the result of insufficient ventral signals which normally repress the organizer [28, 29]. The ADMP effect on the ventral genes is in agreement with the activity of ADMP as part of the BMP morphogenetic network. The organizer-promoting activity is a novel role that can be attributed to ADMP and is in agreement with the expression of this factor within the organizer.

Spatial analysis of the early organizer effect provided the first insight into the endogenous function of ADMP. The reduced organizer-specific gene expression observed in *ADMP* morphants actually translated into a narrower expression area, suggesting that ADMP is required for the organizer to achieve its full domain. Importantly, organizer expansion required low levels of ADMP. In contrast, high ADMP levels reduced the size of the organizer. This role of ADMP in restricting the expansion of the embryonic organizer has been described in zebrafish and chicken embryos [18, 20, 24] and can be interpreted as ventral-expanding phenotypes, a BMP-reminiscent activity. However, our data show that ADMP is a regulator of organizer size that performs opposing functions at slightly different stages [19]. Our analysis suggests that the ventral up-regulation is actually the result of dorsal repression.

*ADMP* and *BMP4* exhibit almost identical kinetics of transcript accumulation during late blastula and early gastrula [19]. Together with the knockdown results, it suggests that early in gastrulation, low ADMP amounts perform the dorsalizing, organizer-promoting function, and as the ADMP amount increases, it crosses a threshold and becomes an anti-dorsalizing (ventralizing) signal. Spatial localization of the ADMP transcripts by whole-mount in situ hybridization (WISH) [16] and qPCR analysis [19] reveal a very tight restriction to the dorsal blastopore lip. Then, any BMP-like function of ADMP requires the diffusion of this protein away from the organizer. Surprisingly, the organizer-restrictive effect of ADMP overexpression was mainly evident when the RNA was injected dorsally and much weaker when delivered ventrally. Similarly, the ventralizing effect of ADMP is stronger when delivered to the dorsal side of the embryo [16]. These observations suggest that either ADMP diffusion is limited or its signaling activity is restricted to the dorsal half of the embryo. Recent studies show that ADMP diffusion proceeds along Brachet’s cleft and the overexpressed ADMP protein is mainly restricted to the dorsal half of the embryo, while the endogenous chordin protein can diffuse throughout the dorsoventral axis [7]. We have previously shown that chordin can facilitate the shuttling of BMP4 [9]. Chordin has also been shown to bind ADMP, and it could facilitate its diffusion [17]. As the diffusion and function of ADMP during early gastrula appear to take place mainly on the dorsal half of the embryo, additional components might play localizing roles like spatial restriction of its receptors.

### Different receptors mediate the dorsal and ventral functions of ADMP

The opposed functions of ADMP could be the result of concentration effects, alternative receptors, post-translational modifications, or co-factors. The increase in the ADMP transcript levels during gastrulation shows that the organizer-expanding function is performed at lower levels than the organizer-restricting role, suggesting a threshold effect, a temporal switch, or the involvement of several receptors. We concentrated on the type I TGFß receptors. Among the receptors, ALK2 binds ADMP, whereas the BMP receptors ALK3 and ALK6 cannot [17]. *Alk2* analysis revealed that this gene is co-expressed with *ADMP* in Spemann's organizer [19]. The *Alk2* expression pattern closely resembles the spatio-temporal pattern of *ADMP* expression in agreement with a functional ligand-receptor connection. Analysis of ALK2 demonstrated that any manipulation hampering the activity of this receptor prevents the organizer from achieving its full size, the same role proposed for ADMP in the establishment of the organizer.

If ALK2 mediates the dorsal, organizer-promoting function of ADMP, another type I TGFß receptor would be expected to mediate the BMP-like, organizer-restricting activity of ADMP. We identified *Alk1* during early gastrula with a temporal pattern closely following the *Alk2* and *Alk3* expression. *Alk1* transcripts are most abundant in the LMZ, while dorsal regions appear not to express this gene [19]. Manipulation of the ALK1 activity revealed an organizer-restricting role, in contrast to the expansion of this domain promoted by ALK2. This ALK1 function coincides with the organizer-restricting, BMP-like role of ADMP. The functional interaction between ADMP and ALK1 was supported by rescue experiments where a dominant negative ALK1 was capable of preventing the organizer restriction induced by ADMP overexpression. Therefore, the opposed regulatory functions of ADMP on organizer size are mediated by two alternative receptors, localized in spatially adjacent regions, mediating opposing responses. Then, cells flanking the organizer are poised to receive an ADMP signal through this receptor.

### ADMP as a regulator of organizer size: a model

**Fig. 8:**
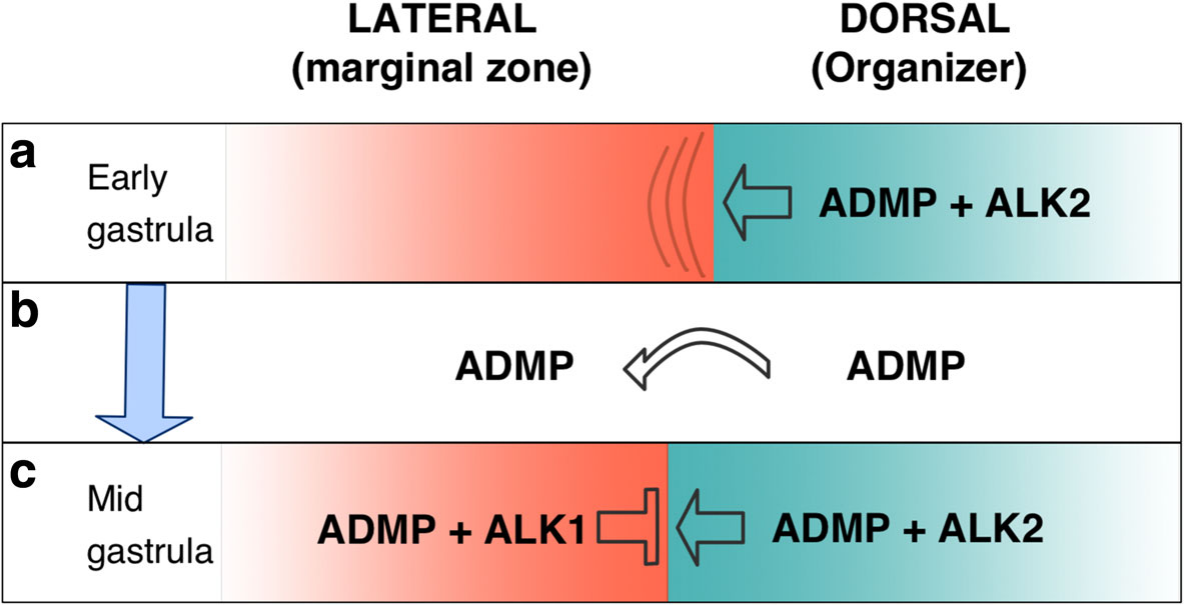
ADMP establishes a self-regulatory network that controls the size of the organizer domain. Model for the ADMP-mediated self-regulatory control of organizer size. **a** During the onset of gastrulation *ADMP* and *Alk2* are already robustly expressed within the organizer. This ligand-receptor pair promotes the enlargement of the organizer and expression of the organizer-specific genes. At this stage, the expression of *chordin* is low. **b** With the progression of gastrulation, the expression of *ADMP* continues to increase, thus allowing the diffusion or shuttling of ADMP to lateral regions flanking the organizer. **c** In the lateral marginal zone (LMZ), the ALK1 receptor is expressed and the ADMP/ALK1 ligand-receptor pair prevents further expansion of the organizer

The results showing the organizer-expanding and organizer-restricting roles of ADMP define a novel self-regulatory mechanism whereby ADMP regulates the size of its own expression domain, and as a result, the overall expression levels of organizer genes. During early gastrula, when ADMP expression is low, it signals through ALK2 performing organizer required function(s) and maintains the size of this domain (Fig. 8a). Loss of this ADMP/ALK2 signal results in a contraction of the organizer domain and the accompanying reduction in organizer-specific gene expression. These observations suggest that ADMP is required for the normal establishment of the organizer domain and the normal expression of organizer genes. As gastrulation proceeds, three events contribute to the reversal of the ADMP activity that actively restricts organizer size. ADMP expression continues to increase until mid-gastrula [16, 17, 19], which results in relatively high levels of ADMP protein within the organizer. This increase in ADMP expression could saturate the available ALK2 receptor and compete with the Nodal signaling within the organizer [22]. The excess protein diffuses to more lateral regions (Fig. 8b) [7].

The second event is the expression of chordin which binds ADMP with a low affinity and antagonizes its activity [7, 17, 21]. Then, chordin may inhibit ADMP from further signaling within the organizer. ADMP molecules that diffuse out of the organizer to more lateral regions might be available for signaling. In principle, the high levels of chordin in the vicinity of the organizer could continue to antagonize the diffused ADMP, but BMPs, and in particular BMP4, in the lateral region might out-compete ADMP for the binding to chordin.

The third component is ALK1 localization in the LMZ. Lateral localization of ALK1 places it in the correct region to receive the ADMP diffusing out of the organizer (Fig. 8c). We have shown that both ADMP and ALK1 have an organizer-repressive activity, and truncated ALK1 rescues the organizer restrictive effect of ADMP overexpression functionally linking this receptor-ligand pair. Then, the laterally localized ADMP/ALK1 signal restricts organizer expansion (Fig. 8c). What prevents this restrictive activity from taking place prematurely? First, it apparently requires higher ADMP levels to saturate the organizer-localized receptors like ALK2 and then to diffuse to lateral regions. Second, *Alk1* shows an expression delay relative to *ADMP* and *Alk2* [19]. We propose that the early absence of ALK1 in the vicinity of the organizer prevents the premature repression of the organizer by increasing ADMP levels. Mathematical modeling of ADMP and its two receptors also supports the conclusion that the localization of ALK1 along the lateral boundary of the organizer prevents further changes in the organizer domain size and it is robust to changes in ADMP levels.

The proposed model involves an intrinsic self-regulatory mechanism to regulate the size of the organizer. By initially supporting the expansion of the organizer, ADMP/ALK2, signaling promotes the expression of early organizer genes including *ADMP* and *chordin*. ADMP then diffuses out of the organizer, and in the LMZ, ADMP/ALK1 signaling restricts the expression of organizer genes. This in turn would also negatively regulate ADMP’s own expression. Regulating the size of the organizer determines the amounts of BMP antagonists secreted by the organizer, which in turn are instrumental in regulating the shape of the dorsoventral BMP morphogenetic and patterning gradient during gastrula. Such a regulatory mechanism would be crucial in adapting the size of the organizer and the BMP gradient to environmental disturbances or embryo size [8, 9].

## Conclusions

In this study, we investigated the function of ADMP during early gastrulation to better understand the apparent conflict between its dorsal expression in the embryonic organizer and its widely accepted role as a repressor of this structure, i.e., its anti-organizer, ventralizing activity. Taking advantage of mainly loss-of-function approaches, we identified at the onset of gastrulation a requirement for ADMP to establish a normal organizer domain. This activity is followed soon thereafter by a reversal in its effect on the organizer, exerting a robust organizer-repressing, anti-organizer effect. Our results identified two type I TGFß receptors that mediate these opposed activities of ADMP. The ACVR1 (ALK2) receptor co-localizes with ADMP within the organizer and mediates the organizer-promoting activity. Reduction in the activity of ALK2, like an early knockdown of ADMP, results in an abnormally small organizer. The second receptor identified, ACVRL1 (ALK1), localizes to lateral regions flanking the organizer and mediates the anti-organizer signal of ADMP. Inhibition of the ALK1 activity, like mid-gastrula reduction of ADMP, results in an expanded organizer. Our results identify a novel role of ADMP in regulating the size of the embryonic organizer domain. We uncovered a self-regulating, expansion-restriction regulatory network where, initially, ADMP with ALK2 promote the establishment of a normal organizer domain. When ADMP diffuses out of the organizer, it binds to ALK1, preventing further changes in organizer size. By regulating the size of the organizer, ADMP affects its own level of expression and also those of many factors secreted from these cells. Among the factors secreted by the organizer are components of the BMP signaling pathway that can shape this morphogenetic gradient to scale it along the dorsoventral axis according to size. A BMP scaling function was previously suggested for ADMP, and the present study provides a mechanistic explanation for this scaling activity.

## Methods

### Embryo culture and treatments

*Xenopus laevis* frogs were purchased from Xenopus I or NASCO (Dexter, MI or Fort Atkinson, WI, USA). Experiments were performed after approval and under supervision of the Institutional Ethics Committee. Embryos were obtained by in vitro fertilization, incubated in 0.1% Modified Barth's Solution and Hepes (MBSH) and staged according to Nieuwkoop and Faber [23]. For stage 10, embryos are selected that begin to have a pigment concentration (small arc) on one side but no groove. At stage 10.25, early invagination (groove) of the pigmented cells appears, with a small arc (~60°). Embryos with extensive invagination along more than 50% of the blastopore and reduction in size of the vegetal plug are considered to be at stage 10.5. For microinjection of capped RNAs or morpholino antisense oligonucleotides, embryos were injected radially (two spaced injections in each blastomere at the two-cell stage or four injections, one in each blastomere, at the four-cell stage) or with one-sided injections (dorsal or ventral). For lineage tracing, the injected material was co-injected with fluorescein isothiocyanate (FITC)-dextran (M_r_ 70,000) 10 pg/injection (Sigma), subsequently detected with anti-fluorescein antibodies (1:5000, Anti-Fluorescein-AP Fab fragments, Roche).

### Complementary DNA (cDNA) clones and constructs

PCR cloning of *Xenopus laevis Alk2* and *Alk1* and mouse *Alk1* was performed using Herculase II Fusion DNA Polymerase (Stratagene, San Diego, CA, USA) using cDNA samples from stages 10.5–11 (*Xenopus*) [19] and multiple stages and organs (mouse). The primers used for cloning are listed in Additional file 4: Table S1. All PCR products were sequenced.

The truncated (dominant negative) mouse ALK1 (tmALK1) and *Xenopus* tALK1 and tALK2 receptors were generated by truncating the intracellular region of the receptors at amino acid position 140 for mALK1, 147 for ALK1, and 143 for ALK2 from *Xenopus laevis*. The primers for PCR amplification of the truncated receptors are listed in (Additional file 4: Table S1); they were used to amplify the right region from reverse transcribed RNA.

### In vitro transcription and gene knockdown

Capped RNA was prepared by in vitro transcription using the iScript cDNA Synthesis Kit (Bio-Rad, Hercules, CA, USA). Cap analog (Pharmacia) was added using a cap:guanosine triphosphate (GTP) ratio of 5:1. The templates for transcription of *Xenopus* tALK2 and tALK1 and mouse tmALK1 mRNA were generated by PCR amplification using a modified version of the preceding primers that included the T7 promoter sequence (5’-TAATACGACTCACTATAGGG) in the forward primers and a poly-A signal sequence (AATAAA) and a stop codon to the reverse primers. The template for tALK3 mRNA was prepared from pSP64TNE BMPR [25]. The template for *Xenopus* ADMP mRNA was prepared from pCR-Script-ADMP [16].

*ADMP* knockdown was induced by injection of a specific antisense morpholino oligonucleotide (ADMPMO) [17]. *Xenopus* *Alk1* and *Alk2* knockdown was induced by injection of the specific antisense morpholino oligonucleotides ALK1MO, 5’-GTTTTCAGGTGACACAGGAGCAGCT-3’ and ALK2MO, 5’-GAAGAATCATAACACCATCCACCAT-3’. A myc-tagged version of the ALK2 protein was constructed by PCR amplification of the open reading frame using the primers 5’-CCCAAGCTTGGGAATGGCTCACTGATTGCAC-3’ and 5’-CGGGATCCCGAACACAGTAATGGGAGAGGCAT-3’ and subcloned into the *Hin*dIII and *Bam*HI sites of the pCS2-MT plasmid.

### Expression analysis

Whole mount in situ hybridization analysis of gene expression was performed as described previously [30]. Probes for in situ hybridization were prepared from the H7 clone for *gsc* [31], ∆59 clone for *chordin* [12], and pCR-Script-ADMP clone for *ADMP* [16].

We performed qPCR using the Bio-Rad CFX384 with C1000 thermal cycler and LightCycler 48 SYBR Green I Master (Roche). All samples were processed in triplicate and analyzed as described previously [32]. All experiments were repeated with at least three different embryo batches. The qPCR primers used are listed in (Additional file 5: Table S2).

### Western blot analysis and protein extraction

Proteins were extracted from embryos (10 embryos per sample) at stage 10.5 using Passive Lysis Buffer (Promega, Madison, WI, USA). Proteins (10–25 μg protein) were resolved on 12% sodium dodecyl sulfate (SDS)-polyacrylamide gel electrophoresis and transferred onto an Immobilon-P Transfer Membrane (Millipore, Bradford, PA, USA). Western blot analysis was performed using anti-myc (9E10), goat anti-mouse peroxidase (POD, Jackson Laboratory, Bar Harbor, ME, USA), and anti-α-tubulin antibodies (Serotec, Oxford, UK) at concentrations of 1:200, 1:10,000, and 1:20,000, respectively. The intensity of the bands was quantitated using the ImageJ software package.

### Mathematical model

The mathematical model describing the interaction between ADMP and its two receptors, ALK1 and ALK2, and the outcome on organizer size is described in Additional file 1: Mathematical model, including a description of the parameters used and the initial values of the parameters employed to run the mathematical model.

### Statistical analysis

All statistical comparisons were carried out using Prism software (GraphPad Software, San Diego, CA, USA). Results are given as the mean ± standard error of the mean (SEM). Tests used were the two-tailed *t* test for two-sample comparisons or Dunnett’s (analysis of variance, ANOVA) multiple comparisons test. Differences between means were considered significant at a significance level of *p* < 0.05.

## Additional files

Additional file 1: Mathematical model. Mathematical model, parameter description and parameter values. (PDF 94 kb)

Additional file 2: Figure S1. Robustness and sensitivity of the size of the organizer induction domain to receptor distribution. (A) Relative organizer induction domain size as a function of ventralization or dorsalization of ALK1 and ALK2 expression at time *t* = 1 h, compared to the reference parameter set. ALK1 and ALK2 expression pattern is given by a Hill function. *org* defines the position along the dorsal-ventral axis where ALK1 and ALK2 reach half their induction. *Dotted line (blue)* denotes the relative organizer size in the reference data set, here, set to 1, which is obtained when *org* equals 500 μm (*black tick*). The relative organizer size is sensitive to the *Alk1*, *Alk2* expression pattern as defined by *org* (400–600 μm). Ventralization of ALK1 and ALK2 expression pattern (*org* < 500) leads to an increase in the organizer induction domain, while dorsalization (*org* > 500) leads to a decrease in organizer size. (B) ALK1 (*red*) and ALK2 (*blue*) profiles for a ventralized parameter (*org* = 450 μm). *Dotted line* shows the position along the dorsoventral axis (*org*) where ALK1 and ALK2 reach half their maximal induction. *Black tick* marks the dorsoventral midline and the position of *org* in the reference parameter set. Each tick in the *X* axis marks 50 μm. (C) Size of the organizer induction domain in the ventralized parameter set as a function of ADMP flux. *Y* axis is the relative size of the organizer induction domain as in A. ADMP flux in reference parameter set is 1 μmnM s^–1^. (D) Profiles (ALK1 (*red*) and ALK2 (*blue*)) for a dorsalized parameter set (*org* = 550 μm). The dorsoventral position where ALK1 and ALK2 reach half their maximal induction, corresponding to *org*, is shown (*dotted line*). (E) Size of the organizer induction domain size in the dorsalized parameter set as a function of ADMP flux as in C. (PDF 240 kb)

Additional file 3: Figure S2. Robustness of the organizer induction domain to several parameters, and dynamics of elements in the model. (A–C) Heat maps for ALK2 occupancy (A), ALK1 occupancy (B), and levels of ADMP (C). *X* axis denotes position along the dorsoventral axis, *Y* axis denotes time, *t*_steady_ is the time when steady state organizer induction is achieved in the dual receptor model. Note that despite the finding that the organizer induction domain is in steady state, ALK1 and ALK2 occupancy, as well as levels of ADMP, are not in steady state. (D–F) Robustness of the organizer induction domain with respect to the ADMP-ALK2 binding rate (D), ADMP-ALK1 binding rate (E), and receptor recycling rate (F). Organizer domain is relative to its size in the reference data set. In the reference data set, ADMP-ALK2 and ADMP-ALK1 binding rate are 0.1 nM^–1^ s^–1^, and receptor recycling rate is 10^–3^ s^–1^. (PDF 432 kb)

Additional file 4: Table S1. Primers used for cloning. (PDF 21 kb)

Additional file 5: Table S2. Primers used for expression analysis (qPCR). (PDF 28 kb)

## Abbreviations

ACVR1: Activin A receptor type I
ACVRL1: Activin A receptor like type I
ADMP: Anti-Dorsalizing Morphogenetic Protein
ALK: Activin receptor-like kinase
BMP: Bone Morphogenetic Protein
LMZ: lateral marginal zone
MO: morpholino antisense oligonucleotide
mRNA: messenger RNA
qPCR: quantitative reverse transcription polymerase chain reaction
tALK: truncated ALK
TGFß: Transforming growth factor beta
