# Supplementary material for: ADMP controls the size of Spemann's organizer through a network of self-regulating expansion-restriction signals
Source: BMC Biol. 2018 Jan 22;16:13. doi: 10.1186/s12915-018-0483-x (PMC5778663; doi:10.1186/s12915-018-0483-x)
Supplement: Supplementary file 1 — Mathematical model. Mathematical model, parameter description and parameter values. (PDF 94 kb) [file 12915_2018_483_MOESM1_ESM.pdf]

Mathematical model

We used a one-dimensional reaction-diffusion model to describe the interactions of ADMP with ALK1 and ALK2 across the dorsal-ventral axis, to gain insight on the formation of the organizer domain.

1) $$\frac{\partial Admp}{\partial t} = D\nabla^2 Admp - \beta \cdot Admp - Admp(k_{A1}Alk1 + k_{A2}Alk2)$$

2) $$\frac{\partial Alk1}{\partial t} = -k_{A1}Admp \cdot Alk1 + r_1 Alk1A$$

3) $$\frac{\partial Alk2}{\partial t} = -k_{A2}Admp \cdot Alk2 + r_2 Alk2A$$

4) $$\frac{\partial Alk1A}{\partial t} = k_{A1}Admp \cdot Alk1 - r_1 Alk1A$$

5) $$\frac{\partial Alk2A}{\partial t} = k_{A2}Admp \cdot Alk2 - r_2 Alk2A$$

Admp, Alk1, Alk2, Alk1A, Alk2A denote ADMP, ALK1, ALK2, ALK1-ADMP complex and the ALK2-ADMP complex. In our model, *Alk1A* and *Alk2A* are the signaling levels by ALK1 and ALK2.

The receptors do not diffuse, and ADMP diffuses with a diffusion coefficient *D*.

β is the ADMP linear degradation rate.

k_{A1} and k_{A2} are the binding rates of ADMP to ALK1 and ALK2 accordingly.

r_1 and r_2 are the rate of ALK1 and ALK2 receptor recycling: the transition from an occupied receptor in complex with ADMP to a free receptor available to interact again with ADMP.

Initial conditions:

1) $$Alk1_{t=0} = Alk1_{min} + \frac{Alk1_{max}}{1+\left(\frac{x}{org}\right)^{h_0}}$$

2) $$Alk2_{t=0} = Alk2_{max}\frac{\left(\frac{x}{org}\right)^{h_0}}{1+\left(\frac{x}{org}\right)^{h_0}}$$

remaining variables are 0 at t=0.

Initial conditions were set to describe high levels of ALK2 and ADMP dorsally, with no expression ventrally. ALK1 is set to have a basal expression in the entire dorsal-ventral axis, with higher abundance at the ventral pole.

$Alk1_{min}$ is the basal level of ALK1 expression throughout the dorsal-ventral axis. $Alk1_{max}$ is the maximal increase in the level of ALK1. *org* is the parameter defining the position where the transition between the basal and maximal ALK1 expression occurs. This transition is given by a Hill function with coefficient h_0 such that if the position *x*, is smaller than the parameter *org*, the initial ALK1 levels approach maximal ALK1 levels, and where *x* is larger than *org*, ALK1 levels approach basal levels. We define the position $x=0$ as the ventral pole of the dorsal ventral axis, and the position $x=L$ as the dorsal pole. For ALK1, the result is high levels at the ventral pole and basal levels at the dorsal pole.

$Alk2_{max}$ is the maximal level of ALK2. The distribution of ALK2 along the dorsal-ventral axis is modeled by a Hill function with a coefficient h_0, where if $x>org$, corresponding to the dorsal region, ALK2 levels are high, while if $x<org$, in the ventral region, ALK2 levels approach zero.

As we write in the main text, the total number of receptors, whether occupied or free is constant throughout the simulation.

Boundary conditions

Boundary conditions are reflective at the ventral pole for all parameters and we furthermore assume a flux η of ADMP from the dorsal pole such that:

$$D\nabla Admp_{x=L} = \eta$$

We note that in our simulations, $Admp_{x=0}=0$ at all times, so the reflective boundary conditions at this pole had no effect.

Organizer induction

Organizer induction was modeled by the following:

$$\theta = \frac{1}{1+\left(\frac{Alk1A}{Thr_1}\right)^h} \frac{\left(\frac{Alk2A}{Thr_2}\right)^h}{1+\left(\frac{Alk2A}{Thr_2}\right)^h}$$

θ is a parameter between 0 and 1 which accounts for organizer induction ($\theta>0.5$) through signaling. It is the product of the inhibition of by ALK1 signaling (*Alk1A*) and induction by ALK2 signaling (*Alk2A*). Thr_1 is the threshold for repression or ADMP by ALK1 signaling, and Thr_2 the threshold for ADMP induction by ALK2 signaling. θ approaches 1 if *Alk1A* is smaller than the repression threshold Thr_1 (little ALK1 signaling), and *Alk2A* is greater than the signaling threshold Thr_2. *h* is the Hill coefficient in this function, where we assumed the same coefficient for both thresholds.

Parameter description

The parameter values for virtually all parameters in this system were not measured directly *in vivo*, We chose the parameter values such that they will fit within physiological range in other systems (www.bionumbers.com), and in accordance with known literature and finally using round numbers to avoid parameter overfitting.

- *D* – ADMP diffusion: ADMP was shown to diffuse rapidly [8].
- k_{A1}, k_{A2} – ADMP and ALK1 or ALK2 association constant: We have no knowledge on the binding rates of ADMP to either receptors, hence we assumed an equal binding rate
- η – ADMP flux: ADMP has a high production rate, evident by the low cycles in the rtPCR reactions as we show in this study.
- β – ADMP degradation rate: We assumed a low degradation rate since it was shown [17] that ADMP can diffuse from the dorsal to the ventral side of the embryo to induce ventral patterning.
- r_1, r_2 - ALK1 and ALK2 receptor recycling rate: In receptor recycling we refer to the rate in which and occupied receptors dissociate from ADMP and are available for signaling again. ADMP is degraded in the process providing a second mode for ADMP degradation. We chose and equal recycling rate for both receptors.
- $Alk1_{min}$, $Alk1_{max}$, $Alk2_{max}$ - ALK1 minimal and maximal concentration, and ALK2 maximal concentration: The values for the initial levels of the receptors set the concentration scale for the entire system, for simplicity we chose the value of 1nM. We set the maximal levels of both receptors to be 1μM as there is no information whether one is more abundant than the other.

- *org* – spatial threshold for ALK1 and ALK2 abundance: This parameter sets the difference between the pre-patterned dorsal-ventral axis. ALK1 is more highly expressed ventrally at $x<org$, and ALK2 is more highly expressed dorsally, where $x>org$ [19]. Changing this parameter changes receptor distribution and affects the position where the organizer is induced. We chose org=0.5mm.
- Thr_2, Thr_1: The threshold for induction and repression were set to allow ADMP induction at half ALK2 receptor occupancy, but high sensitivity for repression by ALK1. These values are arbitrary, and changing these numbers will change the threshold for ADMP induction. We chose the values such that Thr_2 will be lower than the maximal ALK2 occupancy so that ADMP will be induced, and Thr_1 to be higher than the basal level of ALK1 so that ADMP will not be completely repressed through ALK1 signaling at the organizer. We note that increasing the receptor recycling rate reduces the concentration of occupied receptors, hence changing this parameter will change the ADMP induction region. By choosing a slow receptor recycling rate, we simplify the system by having most of the receptors occupied at high ADMP concentration.
- *h* - Hill coefficient *h* for induction and repression of the organizer: This parameter was chosen to be high to account for the sharp threshold seen in *in-situ* mRNA hybridizations for AMDP.
- h_0 - The Hill coefficient for the initial ALK1 and ALK2 distributions: This Hill coefficient was high to account for the distribution of the receptors seen through *in situ* mRNA hybridization.
- *T* - Time of simulation: Time was set for 3600 seconds, which is longer than the approximate time for the developmental process modeled. As our results show, we reach a steady state of the organizer domain in a shorter time.
- *L* – embryo half circumference: Value was set to 1mm keep numbers round setting a higher value would increase the size of the ventral domain. Within the time of simulation, ADMP does not reach the entire dorsal-ventral axis, thus *L* can be made lager without any effect.

Parameter values:

Symbol	*Definition*	*Value*
D	*Admp* Diffusion	10 $\mu m^2 sec^{-1}$
k_{A1}	*Alk1* and *Admp* association rate	10^{-1} $nM^{-1}sec^{-1}$
k_{A2}	*Alk2* and *Admp* association rate	10^{-1} $nM^{-1}sec^{-1}$
η	Admp flux	1 $\mu m nM sec^{-1}$
β	Admp degradation rate	10^{-4} sec^{-1}
r_1	Alk1 receptor recycling rate	10^{-3} sec^{-1}
r_2	Alk2 receptor recycling rate	10^{-3} sec^{-1}
$Alk1_{min}$	Minimal initial Alk1 level	0.1 nM
$Alk1_{max}$	Maximal initial Alk1 level	0.9 nM
$Alk2_{max}$	Maximal initial Alk2 level	1 nM
org	Organizer/Alk2 expression domain	500 μm
Thr_2	Threshold for Admp induction by Alk2-Admp signaling	0.5 nM
Thr_1	Threshold for Admp repression by Alk1-Admp signaling	0.2 nM
h	Hill coefficient	4
h_0	Hill coefficient for Alk1,2 initial level	4
L	Length of dorsal-ventral axis	1000 μm
T	Time of simulation	3600 sec
