# Supplementary figures and images for: ADMP controls the size of Spemann's organizer through a network of self-regulating expansion-restriction signals
Source: BMC Biol. 2018 Jan 22;16:13. doi: 10.1186/s12915-018-0483-x (PMC5778663; doi:10.1186/s12915-018-0483-x)

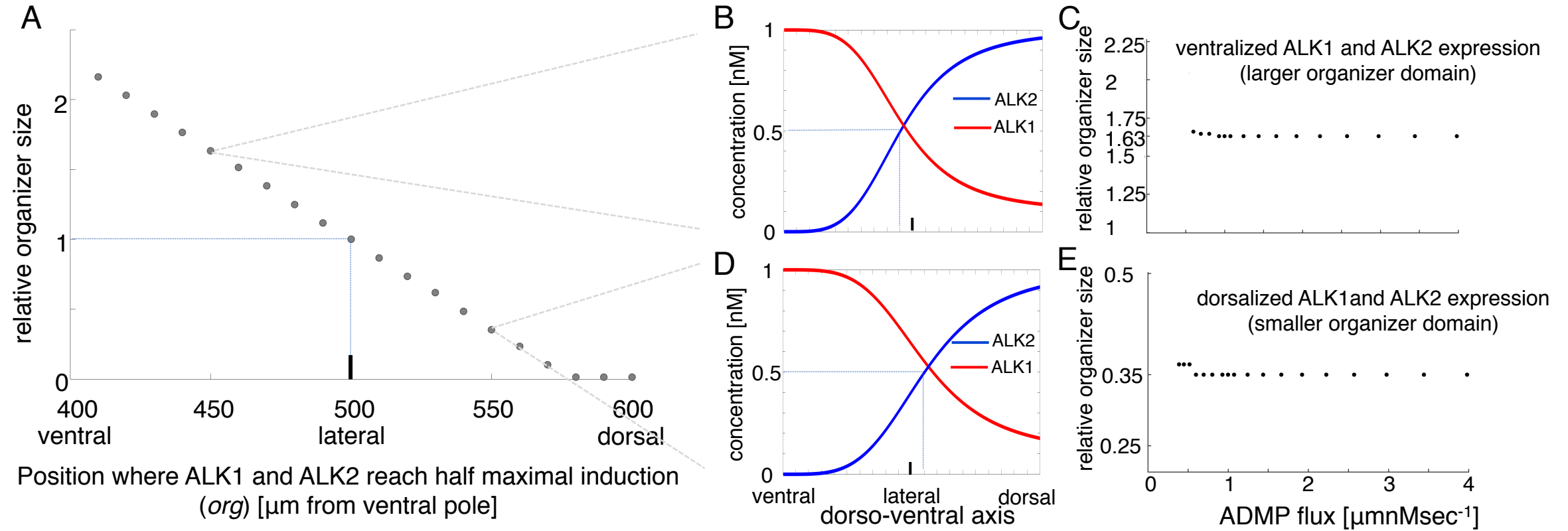

Supplement: Supplementary file 2 — Robustness and sensitivity of the size of the organizer induction domain to receptor distribution. (A) Relative organizer induction domain size as a function of ventralization or dorsalization of ALK1 and ALK2 expression at time t = 1 h, compared to the reference parameter set. ALK1 and ALK2 expression pattern is given by a Hill function. org defines the position along the dorsal-ventral axis where ALK1 and ALK2 reach half their induction. Dotted line (blue) denotes the relative organizer size in the reference data set, here, set to 1, which is obtained when org equals 500 μm (black tick). The relative organizer size is sensitive to the Alk1, Alk2 expression pattern as defined by org (400–600 μm). Ventralization of ALK1 and ALK2 expression pattern (org < 500) leads to an increase in the organizer induction domain, while dorsalization (org > 500) leads to a decrease in organizer size. (B) ALK1 (red) and ALK2 (blue) profiles for a ventralized parameter (org = 450 μm). Dotted line shows the position along the dorsoventral axis (org) where ALK1 and ALK2 reach half their maximal induction. Black tick marks the dorsoventral midline and the position of org in the reference parameter set. Each tick in the X axis marks 50 μm. (C) Size of the organizer induction domain in the ventralized parameter set as a function of ADMP flux. Y axis is the relative size of the organizer induction domain as in A. ADMP flux in reference parameter set is 1 μmnM s–1. (D) Profiles (ALK1 (red) and ALK2 (blue)) for a dorsalized parameter set (org = 550 μm). The dorsoventral position where ALK1 and ALK2 reach half their maximal induction, corresponding to org, is shown (dotted line). (E) Size of the organizer induction domain size in the dorsalized parameter set as a function of ADMP flux as in C. (PDF 240 kb) [file 12915_2018_483_MOESM2_ESM.pdf]

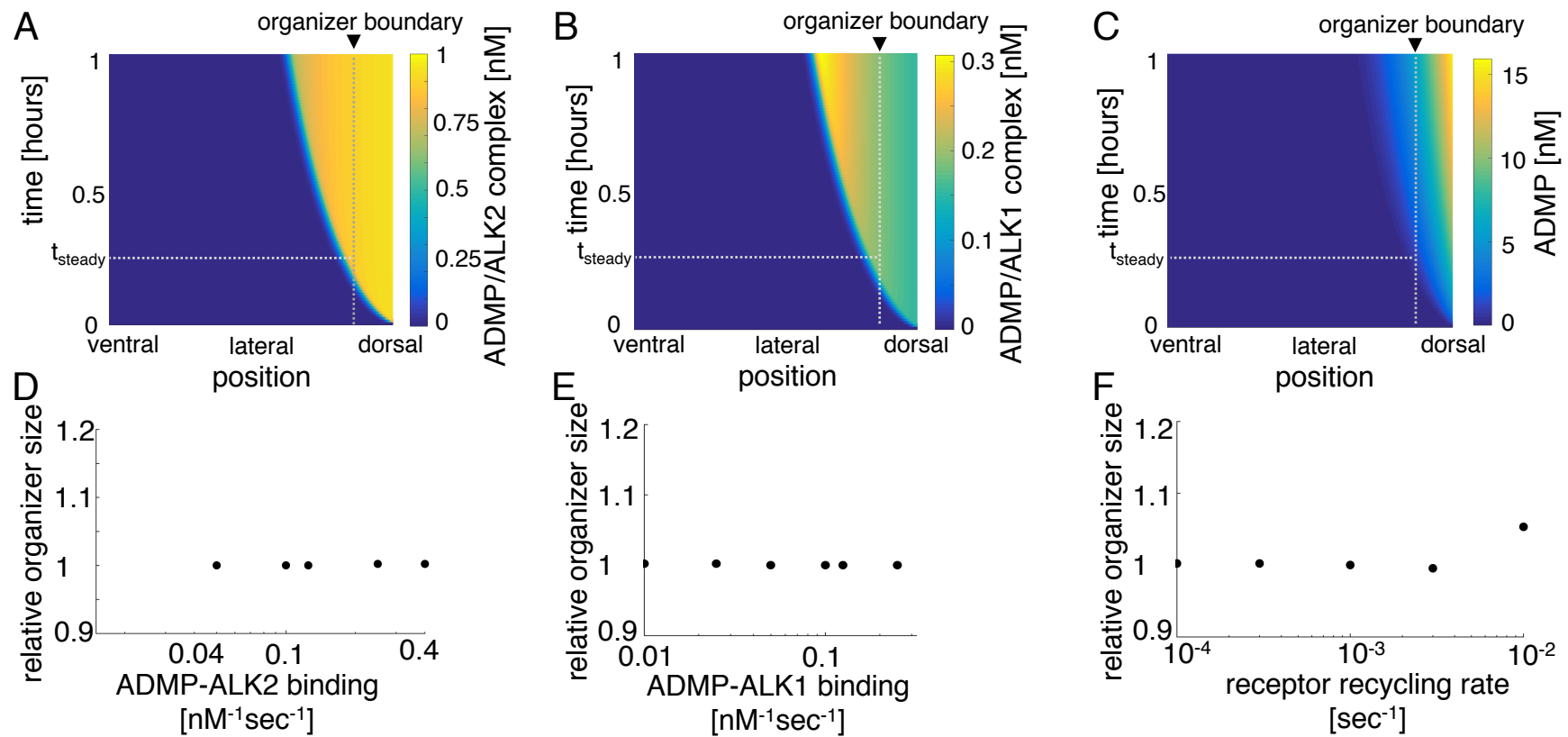

Leibovich et al., Additional File 5: Figure S2

Supplement: Supplementary file 3 — Robustness of the organizer induction domain to several parameters, and dynamics of elements in the model. (A–C) Heat maps for ALK2 occupancy (A), ALK1 occupancy (B), and levels of ADMP (C). X axis denotes position along the dorsoventral axis, Y axis denotes time, tsteady is the time when steady state organizer induction is achieved in the dual receptor model. Note that despite the finding that the organizer induction domain is in steady state, ALK1 and ALK2 occupancy, as well as levels of ADMP, are not in steady state. (D–F) Robustness of the organizer induction domain with respect to the ADMP-ALK2 binding rate (D), ADMP-ALK1 binding rate (E), and receptor recycling rate (F). Organizer domain is relative to its size in the reference data set. In the reference data set, ADMP-ALK2 and ADMP-ALK1 binding rate are 0.1 nM–1 s–1, and receptor recycling rate is 10–3 s–1. (PDF 432 kb) [file 12915_2018_483_MOESM3_ESM.pdf]
