# Supplementary material for: ADMP controls the size of Spemann's organizer through a network of self-regulating expansion-restriction signals
Source: BMC Biol. 2018 Jan 22;16:13. doi: 10.1186/s12915-018-0483-x (PMC5778663; doi:10.1186/s12915-018-0483-x)
Supplement: Supplementary file 4 — Primers used for cloning. (PDF 21 kb) [file 12915_2018_483_MOESM4_ESM.pdf]

Table S1; Primers used for cloning.

Gene	Forward primer	Reverse primer
Alk1 X. laevis	TTCTGTGTCACCTGAAAACCC	GTCTGCCACTTTCATGCCTTT
Alk2 X. laevis	AAGGATCCTGTCCTGCGGAATGG	CAGAATTCCTAACACAGTAATGGGAGAGGC
Alk1 mouse	CACCACAGCTATCACGTTTTGC	CCCTCTACCCAGACAGGCTAT
tALK1	GCGAATTCCAGAGATGATGATA	AGTTTTGCTTTCCAAACCCAGACTATG
tALK2	CGGGATCCCGAGCTGTAAAATGGTGGA	TCCGGAATTCCTACACAGCCAAGACAGA
tmALK1	CACCACAGCTATCACGTTTTGC	GGACACGCCACAAGCCTAGA
