# Supplementary material for: ADMP controls the size of Spemann's organizer through a network of self-regulating expansion-restriction signals
Source: BMC Biol. 2018 Jan 22;16:13. doi: 10.1186/s12915-018-0483-x (PMC5778663; doi:10.1186/s12915-018-0483-x)
Supplement: Supplementary file 5 — Primers used for expression analysis (qPCR). (PDF 28 kb) [file 12915_2018_483_MOESM5_ESM.pdf]

Table S2; Primers used for expression analysis (qPCR).

Gene	Forward primer	Reverse primer
ADMP	GCCTTCCGAGCAAGCTTACTT	CCTTGTGGCAACTGTATCTTATTTTTA
Alk1	GCTCTGGGGAAACTTGTGTT	CAACGCTCCTTTATGCTGTT
Alk2	TGTTATGGGCAGCAGTGTT	GATGTTCAAGTTACAGAGGTCACT
Alk3	TGGCTCAGGGCTACCATTATT	CACCTTCTCTCCTCTCCATTTTC
Alk6	ACAGCAGGAAGGAAGACACA	ACAGTGGTGGTGGCAGTAAC
BMP2	ACACGGACAGCAGAAAACCA	AACAGCAGCAGGAGCAGAGA
BMP4	GCAGCCCAGTAAGGATGT	CTTCTGTGCCTGGTAGATTC
cerberus	CTGGTGCCAAGATGTTCTGGAA	CGGCAAGCAATGGGAACAAGTA
chordin	ACTGCCAGGACTGGATGGT	GGCAGGATTTAGAGTTGCTTC
follistatin	CAGCGACAACACGACTTACC	TCCTCCTCTGTATCTTCAACAATG
GAPDH	GCTCCTCTCGCAAAGGTCAT	GGGCCATCCACTGTCTTCTG
gsc	TTCACCGATGAACAACTGGA	TTCCACTTTTGGGCATTTTC
MyoD	CCCTGTTTCAATACCTCAGACAT	CGTGCTCATCCTCGTTATGG
noggin	CAATGCCAGCGGAAATCA	ATGTGTAAAGGACAGGACAGAAGGT
siamois	CTGTCCTACAAGAGACTCTG	TGTTGACTGCAGACTGTTGA
sizzled	AACAAGGTCTGCTCCTTCCA	CTGTGGGTCTGGTCCGTATC
Ventx1	AGGCAGGAGTTCACAGGAAA	AATGCCTGTTCCAGTTTGCTT
Ventx2	AGAGAGCAGCCAAGCAAAGT	GAAGAAGGGGACACATCACTGT
Ventx3	CCCAGCCAGCACCACAA	AGCATCTTCATCACACACAGGTTT
Wnt8	CTACACCGCAGAGTATTCCA	ATCTCAGGACAGACCAATCG
lhx1	CCCTGGCAGCAACTATGACT	GGGCACAGAGGAAGGTACAA
